# Prevalence and risk factors for depression and anxiety in Chinese patients with Parkinson disease

**DOI:** 10.1186/s12877-017-0666-2

**Published:** 2017-11-22

**Authors:** Shi-Shuang Cui, Juan-Juan Du, Rao Fu, Yi-Qi Lin, Pei Huang, Ya-Chao He, Chao Gao, Hua-Long Wang, Sheng-Di Chen

**Affiliations:** Department of Neurology & Co-innovation Center of Neuroregeneration, Ruijin Hospital Affiliated to Shanghai Jiao Tong University School of Medicine, 197 RuijinEr Road, Shanghai, 200025 People’s Republic of China

**Keywords:** Parkinson disease, Depression, Anxiety, Prevalence, Risk factors

## Abstract

**Background:**

Anxiety and depression are common in Parkinson disease and both are important determinants of quality of life in patients. Several risk factors are identified but few research have investigated general and Parkinson’s disease (PD)-specific factors comprehensively. The aim of this work was to explore PD-specific and -non-specific risk factors for PD with depression or anxiety.

**Methods:**

A cross-sectional survey was performed in 403 patients with PD. Multivariate logistic analysis was used to investigate the prevalence and risk factors for the depression and anxiety in PD. The data of patients included demographic information, medicine history, disease duration, age at onset (AAO), family history, anti-parkinsonism drug, modified Hoehn and Yahr staging (H-Y) stage, scales of motor and non-motor symptoms and substantia nigra (SN) echogenic areas.

**Results:**

403 PD patients were recruited in the study. Depression and anxiety were present in 11.17% and 25.81% respectively. Marital status, tumor, higher Movement Disorder Society-sponsored revision of the Unified Parkinson’s Disease Rating Scale (MDS-UPDRS) II score, dyskinesia, higher Hamilton Anxiety Rating Scale (HARS) score and lower the Parkinson’s disease sleep scale (PDSS) score were associated with depression in PD. female gender, higher rapid eye movement behavior disorder Questionnaire-Hong Kong (RBD-HK) score, higher Hamilton Deprssion Rating Scale (HAMD) score, higher the scale for outcomes in PD for autonomic symptoms (SCOPA-AUT)score and larger SN echogenic areas were associated with anxiety. Neither depression nor anxiety was related to any anti-parkinsonism drugs.

**Conclusions:**

The prevalence of depression and anxiety in the current PD patients was 11.17% and 25.81% respectively. Disease of tumor, currently having no partner, severer motor function, dyskinesia, poorer sleep quality and anxiety were risk factors for PD with depression. Female, depression, rapid eye movement behavior disorder (RBD), autonomic dysfunction and larger SN area were risk factors for PD with anxiety.

## Background

Mood disorders including anxiety and depression are common in Parkinson disease (PD). The average estimated prevalence of depression and anxiety by systematic reviews was ranging from 2.7–90% and 6–55% in PD [1, 2] and both are important determinants of quality of life in patients [3, 4]. However, few studies in China exploring prevalence of mood disorders. Therefore, It is important to explore the prevalence of mood disorder among Chinese PD patients and understand which factors contribute to the development of these symptoms.

Several risk factors specific and not specific to PD for depression and anxiety in PD have been identified. PD-specific factors for depression in PD included more severe motor symptoms, longer disease duration, more advanced disease stage, higher daily levodopa equivalent dose, and the presence of non-motor symptoms such as cognitive decline or sleep disturbances [5–8]. Compared with depression, factors associated with anxiety were less understood in PD [9]. PD-specific risk factors for anxiety in PD included presence of motor fluctuations, depression and dysautonomia [4, 10–12]. In general population, age, gender, educational attainment, marital status and exposure to pesticides and other chemicals, lifestyle such as consumption of tea, coffee, cigarette or alcohol and comorbidities were reported to be related to either mood disorder or PD [13–17] and thus the general factors above may play a role in the development of depression or anxiety in PD. Age, gender, prior depression and anxiety history, family history of depression and anxiety, social support substance dependence and conduct disorder have been considered as risk factors for depression and anxiety for PD in the published articles [8, 11, 18, 19]. And some of these studies indicated that nonspecific general population risk factors are more important markers for anxiety and depression than PD-specific risk factors [8, 11]. However, compared to PD-specific factors, few studies have investigated general factors comprehensively combined with specific factors. Furthermore, to our best knowledge, no study has explored risk factors for depression and anxiety among Chinese population with PD.

Although anxiety and depressive symptoms frequently coexist in PD patients, it remains unclear whether the two symptoms share common or different underlying mechanisms. Few studies have investigated both depression and anxiety and some of studies found they are not linked to the same features of PD [20, 21]. The pathophysiology of anxiety and depression in PD patients still need to be elucidated. Depression is related to severe motor dysfunction and reduced dopamine transporter (DAT) activity in previous study [21, 22]. Previous research suggests anxiety in PD may be partially explained as a psychological respond to the development of disabling motor and non-motor symptoms. Besides, increasing evidence indicated anxiety disorders were related to the neurochemical changes in PD. The noradrenergic and serotonergic systems are thought to be involved in the neurobiology of anxiety in PD [23]. But the presence of anxiety when wearing-off and a positive effect of dopaminergic treatment on anxiety may suggest that the dopaminergic system is involved in the development of anxiety as well [12, 24]. These findings implied the mechanism in depression and anxiety may be in differences to some extent.

Consequently, we hypothesize that some of PD specific and non-specific factors increase risks for depression and anxiety in PD and the risk factors for depression and anxiety are partially different. The aim of our study is to describe the prevalence of depression and anxiety in patients with PD, to explore the risk factors including nonspecific general factors and PD-specific factors for depression and anxiety, and to provide evidence in favor of the hypothesis that anxiety and depression might or might not share common mechanisms in PD.

## Methods

### Participant

The participants in our study were enrolled between Dec 1, 2015, and Dec 31, 2016 from Movement Disorders Clinic at the Department of Neurology, RuiJin Hospital affiliated to Shanghai Jiao Tong University School of Medicine. All patients were diagnosed with idiopathic PD by movement disorders specialists, according to the criteria of Movement Disorder of Society [25]. Patients clinically diagnosed PD aged between 30 and 90 years old and of H-Y stage between 1 and 4 were eligible. Exclusion criteria included deep brain stimulation (DBS), secondary Parkinsonism and atypical Parkinsonism, other movement disorders other than PD, severe hearing or visual loss, inability to speak or write, or other conditions that might interfere with the reliable completion of clinical assessments. The study was approved by the medical ethics committee of Rui Jin Hospital affiliated to Shanghai Jiao Tong University School of Medicine. Participants gave written informed consent before inclusion in the study.

### Assessment

All information was collected by a face-to-face interview during one-time interview in outpatient clinics. Depression and anxiety were quantified with the 17-item Hamilton Depression Rating Scale (HAMD) and Hamilton Anxiety Rating Scale (HARS), respectively. The optimal threshold to utilize for maximum discrimination between depressed and non-depressed PD patients was reached at a cut-off score of 13/14 for the HAMD and 12/13 for HARS [26, 27]. Additionally, because of the potential improvement of depressive and anxious symptoms due to the anti-depression treatment, participants who were taking medication specifically for depression and anxiety were directly considered as depression and anxiety and they were all evaluated by HAMD and HARS.

PD-non-specific data includes demographic information, exposure, lifestyle factors. Demographic information including age, sex, educational attainment and marital status was collected during a clinical interview. Marital status was classified as either currently having or not having a life-partner. Exposures to pesticides and other chemicals, consumption of tea and coffee, smoking and alcohol status, physical activity and disease information were studied. Regular exposure was defined as “having used pesticide, heavy metal and aerosol weekly for a period of 6 months or more.” Lifestyle factors were shown as categorical factors according to previous studies. Tea and coffee drinkers were defined as “consuming more than 1 cup of tea of coffee” as a previous study found that individuals with more than 1 cups daily was associated with reduced risk for PD [28]. Patients with exercise habit were defined as “moderate or vigorous physical activity at least once weekly” [29]. Ever smoker was identified by using question “whether they had ever smoked at least one cigarette a day for 1 year or longer” [30]. Ever drinker is defined as “drank an average of at least one glass of alcohol per week” [31]. History of vascular risk factors, autoimmune diseases, stroke, tumor and peptic ulcer were also studied.

PD-specific variables including age at onset (AAO), disease duration, family history of PD and current anti-parkinsonism medications were collected. The equivalent daily dose of L-dopa (mg/day) of dopamine agonists, Catechol-O-methyltransferase (COMT) and Monoamine oxidase (MAO-B) inhibitors was calculated for each patient as previously proposed [32]. Disease stage was assessed with the Hoehn Yahr staging (H-Y stage). Motor function, disease-related decline in ADL, and complications of therapy were assessed with the Movement Disorder Society Unified Parkinson’s Disease Rating Scale (MDS-UPDRS). Patients were also classified according to whether they reported freezing of gait (FOG) (MDS-UPDRS Part II item 13 > 1), fluctuation (MDS-UPDRS Part IV item 3 ≥ 1) and dyskinesia (MDS-UPDRS Part IV item 1 ≥ 1). Rate of disease progression was estimated from cross-sectional data by dividing the MDS-UPDRS III total score by duration of disease in years and dichotomized on the sample median [20].

Cognitive function was assessed with the MMSE (mini-mental state examination). Olfactory function was assessed with 16-item odor identification test from the extended version of sniffin’ sticks (SS-16) [33]. autonomic function was assessed with the scale for outcomes in PD for autonomic symptoms (SCOPA-AUT). Sleep quality, rapid eye movement behavior disorder(RBD), and excess daytime sleep were assessed with the Parkinson’s disease sleep scale (PDSS), the RBD Questionnaire-Hong Kong (RBD-HK) and Epworth Sleepiness Scale (ESS). Fatigue and pain were assessed with The Fatigue Severity Scale (FSS) and Brief Pain Inventory (BPI). transcranial sonography(TCS) data for the larger substantia nigra (SN) echogenic areas was collected [34].

### Statistical analysis

Statistical analyses were performed with SPSS Statistics (version 20.0, SPSS Inc., Chicago, IL, USA). Continuous variables were given as means. Categorical variables were summarized by percentages. We used chi square tests for categorical variables, t tests for normally distributed variables and Mann-Whitney tests for non-parametric variables for between-group comparisons. Logistic regression analysis was used to identify possible risk factors for depression and anxiety. As sex and age may have influence on both dependent and independent variables, the univariate relationship adjusted by sex and age between depression or anxiety and each predictor variable was examined. Any variables that had univariate associations with *P* values less than 0.20 were included in a multivariate model, also adjusting for age and sex. A backward selection approach was used to choose the best model [21]. Variables were removed one at a time until all variables remaining in the model were significant at the 0.10 level, which is common in a selection procedure for a prediction model [35].

Logistic regression models were performed to determine whether antiparkinsonian drugs were correlated with depression and anxiety adjusted for age, sex, H-Y stage and the severity of anxiety or depressive symptoms assessed by HARS or HAMD.

Risk factors were calculated as odd ratios (OR) with 95% confidence intervals (CI). Significance of differences was defined as two-tailed *p* < 0.05.

## Results

403 patients participated in this study. The information regarding all patients were shown in Table 1. Depression and anxiety were present in 11.17%(*n* = 45) and 25.81% (*n* = 104) of our sample respectively.9.73%(*n* = 39) had both depression and anxiety. Five patients were taking anti-depression medication.

**Table 1:** Characteristics of the patients with Parkinson’s disease with or without depression or anxiety

	Total patients (*N* = 403)	Patients with depression (N = 45)	Patients without depression (*N* = 358)	*P* value	Patients with anxiety (*N* = 104)	Patients without anxiety (*N* = 299)	*P* value
Age, years‡	62.6	65.2	62.2	0.097	65.4	61.6	0.002
Men (%)^*^	55.4	48.8	56.1	0.362	51.5	56.7	0.355
Currently having no partner (%)^*^	5.3	14.3	5.2	0.096	9.4	5.1	0.485
Schooling year <9(%)^*^	18.0	29.5	16.6	0.035	24.3	15.9	0.056
Regular exposure (%)^*^	23.7	23.0	29.3	0.374	28.0	22.2	0.240
Vascular disease (%)^*^	39.8	47.7	38.7	0.248	43.7	38.3	0.336
Autoimmune disease (%)^*^	4.5	9.1	4.0	0.247	4.9	4.4	0.856
Stroke (%)^*^	3.5	2.3	3.7	0.635	7.8	2.0	0.016
tumor (%)^*^	1.5	4.5	1.1	0.0080	1.0	1.7	0.601
peptic ulcer (%)^*^	3.5	6.8	3.1	0.208	8.7	1.7	0.002
Having smoking (%)^*^	18.1	25.0	17.3	0.210	19.4	17.7	0.695
Having drinking (%)^*^	13.1	15.9	12.7	0.558	12.6	13.3	0.868
Exercise (%)^*^	53.0	39.5	54.7	0.060	44.1	56.2	0.036
Tea (%)^*^	22.6	12.2	23.9	0.092	14.0	25.6	0.017
Coffee (%)^*^	8.5	4.9	8.9	0.562	8.0	8.7	0.841
Age at onset‡	57.1	57.8	57.0	0.758	57.9	56.8	0.358
Duration‡	5.5	7.5	5.3	0.005^*^	7.5	4.9	< 0.001
H-Y stage‡	1.9	2.5	1.8	< 0.001	2.3	1.8	< 0.001
Family history‡	16.5	12.2	17.0	0.429	11.9	18.1	0.145
MDS-UPDRS II‡	12.1	19.0	11.2	< 0.001	17.4	10.2	< 0.001
MDS-UPDRS III‡	27.9	38.1	26.6	0.001	37.2	24.6	< 0.001
Dyskinesia (%)^*^	5.5	13.3	4.5	0.034	10.6	3.7	0.008
Fluctuation (%)^*^	19.6	31.1	18.2	0.039	32.7	15.1	< 0.001
Fast progression (%)^*^	50%	42.2	51.5	0.238	48.1	51.4	0.566
FOG (%)^*^	15.9	33.3	13.7	0.001^*^	34.6	9.4	< 0.001
MMSE‡	26.6	25.4	26.8	0.011	25.3	27.1	< 0.001
PDSS‡	115.7	94.4	118.4	< 0.001	100.7	120.9	< 0.001
HARS/HAMD‡	9.5/7.1	20.4	8.0	< 0.001	12.6	5.1	< 0.001
BPI‡	13.0	25.4	11.4	< 0.001	21.4	10.0	< 0.001
SS-16‡	7.1	6.2	7.2	0.074	6.4	7.4	0.015
RBD‡	17.8	23.5	17.0	0.019	24.0	15.7	< 0.001
SCOPA-AUT‡	12.9	22.4	11.6	< 0.001	21.2	10.0	< 0.001
FSS‡	32.2	44.0	30.6	< 0.001	40.1	29.3	< 0.001
ESS‡	7.3	8.7	7.1	0.075	9.1	6.7	0.001
SN echogenic areas‡	11.3	11.5	11.2	0.831	11.8	10.9	0.645

*chi square test. ‡ Non-parametric statistics (Mann-Whitney test). H-Y stage: modified Hoehn and Yahr staging; MDS-UPDRS: Movement Disorder Society-sponsored revision of the Unified Parkinson’s Disease Rating Scale; FOG: freezing of gait; MMSE: mini-mental state examination; PDSS: the Parkinson’s disease sleep scale; HAMD: 17-item Hamilton Depression Scale; HARS: item Hamilton Anxiety; RBD-HK: the RBD Questionnaire-Hong Kong; SCOPA-AUT: the scale for outcomes in PD for autonomic symptoms; ESS: Epworth Sleepiness Scale; FSS: The Fatigue Severity Scale; BPI: Brief Pain Inventory SN: substantia nigra

Adjusted for age and sex in the univariate model, depression was associated with history of depressive or anxious disease, younger AAO, longer disease duration, more advanced H-Y stage, higher score of MDS-UPDRS II and MDS-UPDRS III, existence of dyskinesia, motor fluctuation and FOG, lower score of MMSE, PDSS and SS-16 and higher score of HARS, BPI, RBD-HK, FSS, SCOPA-AUT and ESS (Table 2). Non-specific variables associated with anxiety in PD were age, stroke, history of depressive or anxious disease and peptic ulcer. PD specific variables associated with anxiety were AAO, disease duration, H-Y stage, MDS-UPDRS II, MDS-UPDRS III, dyskinesia, fluctuation, FOG and non-motor symptoms including cognition, sleep, anxiety, RBD, pain, odor, daytime sleep, autonomic function and fatigue (Table 2).

**Table 2:** Relationship between depression or anxiety with demographics, clinical characteristics in PD subjects by logistic regression analysis adjusted for age and sex

	Depression		Anxiety	
	Adjusted OR (95% CI) for depression in PD	*P* value	Adjusted OR (95% CI) for anxiety in PD	*P* value
Age, years	1.031 (0.998–1.065)	0.065*	1.041(1.017–1.066)	0.001*
Men	0.830 (0.442–1.559)	0.562	0.845 (0.534–1.335)	0.469
Currently having no partner	2.437 (0.855–6.950)	0.096*	1.386 (0.555–3.461)	0.485
Schooling year <9	1.806 (0.865–3.771)	0.116*	1.512(0.856–2.673)	0.154*
Regular exposure	1.272 (0.621–2.603)	0.511	1.340 (0.794–2.263)	0.273
Vascular disease	1.230 (0.639–2.369)	0.536	1.032 (0.641–1.660)	0.897
Autoimmune disease	2.219 (0.675–7.293)	0.189*	0.921(0.309–2.747)	0.883
Stroke	1.187 (0.253–5.562)	0.828	3.373 (1.125–10.108)	0.030*
tumor	4.450 (0.779–25.417)	0.093*	0.600 (0.069–5.251)	0.645
peptic ulcer	2.112 (0.559–7.979)	0.270	5.140 (1.652–15.986)	0.005*
Having smoking	2.341 (0.970–5.647)	0.058*	1.384 (0.724–2.647)	0.326
Having drinking	1.667 (0.636–4.371)	0.298	1.098 (0.528–2.281)	0.803
Exercise	0.577 (0.302–1.102)	0.096*	0.637 (0.401–1.012)	0.056*
Tea	0.467 (0.175–1.246)	0.128*	0.541 (0.290–1.009)	0.053*
Coffee	0.460(0.105–2.011)	0.302	0.907 (0.403–2.044)	0.814
Age at onset	0.926 (0.874–0.980)	0.008*	0.905 (0.864–0.949)	< 0.001*
Duration	1.080(1.020–1.144)	0.008*	1.104 (1.054–1.158)	< 0.001*
H-Y stage	3.338(2.133–5.221)	< 0.001*	2.833 (1.973–4.067)	< 0.001*
Family history	0.715 (0.288–1.778)	0.470	0.666 (0.349–1.273)	0.219
MDS-UPDRS II	1.103 (1.063–1.146)	< 0.001*	1.122 (1.084–1.161)	< 0.001*
MDS-UPDRS III	1.026 (1.010–1.042)	0.001*	1.034 (1.022–1.048)	< 0.001*
Dyskinesia	3.059 (1.109–8.435)	0.031*	2.851 (1.164–6.981)	0.022*
Fluctuation	2.231 (1.111–4.482)	0.024*	3.103 (1.814–5.307)	< 0.001*
Fast progression	1.468(0.773–2.785)	0.240	0.916 (0.580–1.447)	0.707
FOG	2.629 (1.274–5.426)	0.009*	4.400 (2.461–7.867)	0.096*
MMSE	0.919 (0.845–0.999)	0.048*	0.885 (0.828–0.945)	< 0.001*
PDSS	0.958 (0.945–0.972)	< 0.001*	0.965 (0.955–0.975)	< 0.001*
HARS/HAMD	1.343 (1.243–1.451)	< 0.001*	1.610 (1.460–1.775)	< 0.001*
BPI	1.046 (1.027–1.066)	< 0.001*	1.044 (1.028–1.060)	< 0.001*
SS-16	0.919 (0.834–1.012)	0.086*	0.945 (0.882–1.013)	0.112*
RBD	1.019 (1.002–1.036)	0.024*	1.024 (1.011–1.037)	< 0.001*
SCOPA-AUT	1.139 (1.095–1.185)	< 0.001*	1.169 (1.128–1.212)	< 0.001*
FSS	1.036 (1.017–1.055)	< 0.001*	1.031 (1.018–1.044)	< 0.001*
ESS	1.046(0.993–1.103)	0.090*	1.071 (1.030–1.114)	0.001*
SN echogenic areas	0.976(0.938–1.015)	0.288	1.017 (0.991–1.033)	0.110*

*Included in multivariate logistic regression analysis (p < 0·2). H-Y stage: modified Hoehn and Yahr staging; MDS-UPDRS: Movement Disorder Society-sponsored revision of the Unified Parkinson’s Disease Rating Scale; FOG: freezing of gait; MMSE: mini-mental state examination; PDSS: the Parkinson’s disease sleep scale; HAMD: 17-item Hamilton Depression Scale; HARS: item Hamilton Anxiety; RBD-HK: the RBD Questionnaire-Hong Kong; SCOPA-AUT: the scale for outcomes in PD for autonomic symptoms; ESS: Epworth Sleepiness Scale; FSS: The Fatigue Severity Scale; BPI: Brief Pain Inventory SN: substantia nigra

Adjusted for age, sex, H-Y stage and the severity of anxiety or depressive symptoms assessed by HAMD or HARS, no significant correlation was found between patients with depression or anxiety and anti-parkinsonism drug (Table 3).

**Table 3:** Logistic regression model of antiparkinsonian drugs significantly associated with depression and anxiety

Antiparkinsonian treatment	Patients with depression	Patients without depression	Adjusted OR (95% CI)	*P* value	Patients with anxiety	Patients without anxiety	Adjusted OR (95% CI)	*P* value
L-DOPA (mg/d)	385.50	238.70	1.000(0.999–1.002)	0.877	365.72	218.87	1.001(0.999–1.002)	0.266
Dopamine agonist (mg/d)	52.00	41.79	1.002 (0.995–1.009)	0.662	53.12	39.53	1.005 (1.000–1.007)	0.073
MAO-B inhibitor (mg/d)	15.00	18.86	0.993 (0.981–1.005)	0.232	16.8	18.92	1.001 (0.993–1.010)	0.763
COMT inhibitor (mg/d)	10.12	4.22	0.997 (0.984–1.007)	0.607	8.94	3.57	1.003(0.992–1.014)	0.575
Amantadine (mg/d)	44.00	43.86	0.996 (0.992–1.001)	0.106	56.73	39.36	1.003 (1.000–1.006)	0.092

In a logistic regression analysis with depression or anxiety as dependent variable, using as independent variables age, gender and the variables that showed univariate association (*p* < 0·2) with PD with depression or anxiety, we found currently having no partner (OR = 7.616, *p* = 0.014), tumor (OR = 92.206, *p* < 0.001), higher MDS-UPDRS II score (OR = 1.148, *p* = 0.006), dyskinesia (OR = 5.944, *p* = 0.048), higher HARS score (OR = 1.358, *p* < 0.001) and lower PDSS score (OR = 0.959, *p* = 0.001) were risk factors for depression in PD (Table 4), while female gender (OR = 0.284, *p* = 0.026), higher RBD-HK (OR = 1.029, *p* = 0.037), higher HAMD score (OR = 1.697, p < 0.001), higher SCOPA-AUT score (OR = 1.146, p < 0.001) and larger SN echogenic areas (OR = 1.034, *p* = 0.030) were risk factors for anxiety in PD (Table 5).

**Table 4:** Model for prediction of depression in patients with Parkinson’s disease

	Odds ratio (95% CI)	*P* value
Marital status	7.616(1.497–36.816)	0.014
tumor	92.206(7.616–1116.279)	< 0.001
MDS-UPDRS II	1.148(1.040–1.267)	0.006
Dyskinesia	5.944(1.013–34.878)	0.048
PDSS	0.959(0.935–0.983)	0.001
HARS	1.358(1.218–1.515)	< 0.001

Backward results of multivariate logistic regression with all statistically significant variables. MDS-UPDRS: Movement Disorder Society-sponsored revision of the Unified Parkinson’s Disease Rating Scale; PDSS: the Parkinson’s disease sleep scale; HARS: item Hamilton Anxiety

**Table 5:** Model for prediction of anxiety in patients with Parkinson’s disease

	Odds ratio (95% CI)	*p* value
sex(male: female)	0.284(0.094–0.861)	0.026
HAMD	1.697(1.423–2.025)	< 0.001
RBD-HK	1.029(1.002–1.057)	0.037
SCOPA-AUT	1.146(1.072–1.224)	< 0.001
SN echogenic areas	1.034(1.003–1.066)	0.030

Backward results of multivariate logistic regression with all statistically significant variables. HAMD: 17-item Hamilton Depression Scale; RBD-HK: the RBD Questionnaire-Hong Kong; SCOPA-AUT: the scale for outcomes in PD for autonomic symptoms; SN: substantia nigra

## Discussion

This study is the first to explore the prevalence and PD specific and non-specific predictor for both depression and anxiety comprehensively in Chinese PD patients of large sample size. In our study, PD patients with depression accounted for about 11.17% and anxiety for 25.81% based on scales, much higher than those reported in the general population [36], consistent with previously reported prevalence rates of depression and anxiety in PD [1, 2]. The prevalence for depression and anxiety in PD in previous studies varied widely attributed to different population enrolled in research, different rating scales and different types of depressive and anxious disorders included [1, 2]. Few studies explored the prevalence of depression and no study explored the prevalence of anxiety among Chinese PD patients of a large sample. Chan P, et al. enrolled 1047 Chinese sporadic PD cases and found 19.8% patients had depression [37]. The discrepancy may come from different scales used to screen depression. In our case, most patients with depression (39/45) coexisted with anxiety and depression alone was only observed in 6 patients accounting for about 1.5%. A previous study from Taiwan also found that depression alone was only 2.2% in PD patients assessed by self-reported Beck Depression Inventory (BDI) and Beck Anxiety Inventory (BAI) [38]. S. Landau et al. classified PD into four groups: ‘high anxiety + depression’, ‘moderate anxiety + depression’, ‘anxiety’, ‘psychologically healthy’, by interpretation of the latent transition analysis (LTA) model, instead of a class with a profile characterized by predominantly depressive symptoms [39]. Increased risk for anxiety in depression both with and without PD was found in previous studies and the rate of comorbid was higher in PD than that in general population [40–42]. The tripartite model developed by Clark and Waston, in which general factors are assumed to exist in both depression and anxiety as shared general distress, as well as specific factors characterizing the distinct aspects of anxiety and depression, may explained it [42]. The prevalence of a co-morbid depression disorder in patients with anxiety is 36%, slightly higher than previous studies.

Six predictors are identified for depression in PD including history of tumor and marital status, MDS-UPDRS II score, dyskinesia, HARS score and PDSS score. Scant studies have explored the association between marital status and depression in PD. But currently not married or not living with spouse are reported to be associated with elevated risk of depression assessed by Geriatric Depression Scale (GDS) among general population in a study in China [16]. Cancer was reported to be associated with depression in previous studies and these two may share biobehavioral mechanisms including inflammation and oxidative/nitrosative stress [13, 43, 44]. More severe motor symptoms have been commonly identified as the predictor of depressive symptoms [18, 21, 45]. Our study confirmed the results with the statistical significance. However, we failed to find the association between MDS-UPDRS part III and depression. The inconsistency may result from the influence of medicine as all patients were in the “on” phase and stronger perception of disability than actual disability in depressed PD [46]. A positive association was observed between dyskinesia and depression in PD in our study that was addressed in past few literature [6, 45, 47]. In one study based on animal model, the antidepressant selective serotonin reuptake inhibitors (SSRIs) are able to fully counteract levodopa-induced dyskinesia (LID) in 6-OHDA-lesioned rats, implying the associations between the two symptoms [48].Besides, patients with dyskinesia are more likely to have a more severe motor symptoms [49]. In line with previous studies [6, 45, 50], worse sleep disturbance was found to be a risk for depression in PD in our study. Changes in neurotransmitters may contribute to the association including acetylcholine, serotonin and noradrenalin [50, 51].

Five variables are identified as predictor for anxiety in PD including sex, HAMD score, RBD-HK score, SCOPA-AUT score and SN echogenic areas in this study. In accordance with those previously reported in general non-parkinsonian populations [17], female was also considered an established risk factor for anxiety in patients with PD [11, 12, 52, 53]. This finding may account for biological factors (such as hormonal changes), psychological and social factors such as greater stress when encountering life’s adversities like PD [17]. Our study identified dysautonomia as a risk factor for anxiety in PD. An association between anxiety and dysautonomia in PD was found in earlier studies. Jiang SM, et al. compared dysautonomia in 99 patients with and without clinically relevant anxiety by HARS and the Non-Motor Symptoms Questionnaire (NMSQT) and found urinary disorder was the factors for anxiety in PD [54]. A longitude study in Europe also found an association between anxiety and dysautonomia [53]. The role of central noradrenergic dysregulation may play a role in both dysautonomia and anxiety [55]. Additionally, autonomic failure may create a pathophysiological predisposition towards the somatic symptoms of anxiety or a stronger physical response to anxiety in PD, as reported in previous research [56]. Subjects with higher RBD-HK scores was a risk factor for anxiety in PD in the study, which is in line with previous studies [35, 57]. A potential explanation for this finding is a more diffuse neurodegenerative process in the serotoninergic raphe and other brainstem nuclei in PD subjects with RBD [57, 58]. The finding of larger SN echogenic areas as a prominent factor for anxiety was a novel result of this study that to our best knowledge have not been reported in the literature. We speculate this finding may be explained by iron load. Published studies indicated that iron overload appears to alter anxiety-like behavior and mood [59, 60]. Berg et al. found iron may lead to an increase of echogenicity of the SN [61]. This association needs to be confirmed by larger sample and mechanism underlying needs to be explored further.

Unlike previous studies [6, 18, 21, 35, 45, 53, 62], the present study failed to find association between cognition and mood disorder. Low sensitivity to detect cognitive decline of MMSE partially accounts for the irrelevance. And the patients recruited in our study were mainly early stage of PD, leading to relatively slight cognitive impairment that was hardly distinguished by MMSE.

In the study, anxiety and depression were co-morbid and both were the risk factor for each other. This can be considered as an argument to support the hypothesis that these two symptoms share common pathophysiological mechanisms. However, depression can be present in the absence of anxiety and vice versa. Except some general factors related to oxidative stress and inflammation. PD with depression and anxiety in our study have different predictors respectively. Depressive symptoms were mainly associated with indices of PD severity (MDS-UPDRS score, dyskinesia) except sleep disturbance, whereas clinical factors most strongly associated with anxiety were a complex of extra-nigral non-motor symptoms (dysautonomia and RBD) that do not improve with dopaminergic treatment, referred as predominantly non-dopaminergic (PND) features. We speculate that depression and anxiety are co-morbid but partially dissociable. Depression was related to a complex combination of dopaminergic and non-dopaminergic perturbations and dopaminergic dysfunction takes most responsibility, which was proved by previous biomarker and neuroimaging studies [21, 23, 63, 64]. Anxiety was related mainly to non-dopaminergic pathology, which was proved by the finding that striatal DAT-binding ratio was not found to be significantly associated with anxiety in a previous study [35]. A lack of correlation between the consumption of any antiparkinsonian drugs and anxiety in our study and published literature also supports our speculation [21]. Several studies published also found the different predictor for depression and anxiety similar with our study, reinforcing our hypothesis [21, 53, 65]. These hypotheses need to be explored by molecular neuroimaging including dopamine, serotonin and other neurotransmitters.

The present results had some novelty and clinical relevance. First, this study added information about prevalence and risk factors of depression and anxiety among Chinese PD patients enrolled a large sample of patient. Secondly, previous prospective studies about mood disorders were lack of information about PD non-specific factors. The broad variables including PD-specific and non-specific factors in the present cross-sectional study are useful to guide the evaluation of risk factors for depression and anxiety in PD. Furthermore, the results showed no association between anxiety and motor symptoms, thus indicating that anti-parkinsonism medication may have no effects on anxious symptoms. Additionally, the reported factors implied depression and anxiety were multifactorial.

The results of the present study must be considered in light of its limitations. First, the diagnosis of depression and anxiety is based on scales instead of DSM-V criteria. Due to an overlap of symptoms of depression or anxiety and PD, one may argue that it may lead to inaccuracy and overestimation. However, we attempted to control this potentially distorting effect on our results using a PD-specific cutoff value for depression and anxiety. It’s reported that the sensitivity of HAMD is 88% and specificity is 89%, and HARD is 75% and 70% in PD [26]. HAMD was recommended for use by the Movement Disorders Society to screen for symptoms of depression [26], and HADS was classified as ‘suggested’ for assessment of anxiety in PD by a Movement Disorder Society task force [66]. Secondly, drugs may have influence on the result of assessment of motor symptom. However, we still found significant differences in motor function between depressed and non-depressed PD patients. The differences can be exaggerated without medicine. Besides, some variables possibly related to mood disorders were not included in the study, such as adversities and personality. Furthermore, MMSE was used to assess cognition in the study. This scale has low sensitivity to detect cognitive decline in PD. More sensitive scale will be needed in our further study. Last but not the least, the study is cross-sectional and has limitation in reflecting whether these risk factors contribute to depression or anxiety or the latter influence these variables. But as we mentioned above, this cross-section study may help to screen factors in longitudinal study.

## Conclusion

In summary, the prevalence of depression and anxiety in the current PD patients was 11.17% and 25.81% respectively. Tumor, current having no partner, severer motor function, dyskinesia, poorer sleep and anxiety were risk factors for PD with depression. Female, depression, RBD, autonomic dysfunction and larger SN area were risk factors for PD with anxiety.

## Abbreviations

AAO: Age at onset
BAI: Beck Anxiety Inventory
BDI: Beck Depression Inventory
BPI: Brief Pain Inventory
CI: Confidence intervals
COMT: Catechol-O-methyltransferase
DAT: Dopamine transporter
ESS: Epworth Sleepiness Scale
FOG: Freezing of gait
FSS: The Fatigue Severity Scale
GDS: Geriatric Depression Scale
HAMD: Hamilton Depression Rating Scale
HARS: Hamilton Anxiety Rating Scale
H-Y stage: Modified Hoehn and Yahr staging
LID: Levodopa-induced dyskinesia
LTA: Latent transition analysis
MAO: Monoamine oxidase
MDS-UPDRS: Movement Disorder Society-sponsored revision of the Unified Parkinson’s Disease Rating Scale
MMSE: Mini-mental state examination
NMSQT: the Non-Motor Symptoms Questionnaire
PD: Parkinson’s disease
PDSS: the Parkinson’s disease sleep scale
PND: Predominantly non-dopaminergic
RBD: Rapid eye movement behavior disorder
RBD-HK: the RBD Questionnaire-Hong Kong
SCOPA-AUT: the scale for outcomes in PD for autonomic symptoms
SN: substantia nigra
